# Combined Ki67 and ERCC1 for prognosis in non-keratinizing nasopharyngeal carcinoma underwent chemoradiotherapy

**DOI:** 10.18632/oncotarget.19158

**Published:** 2017-07-11

**Authors:** Ying Lu, Haixin Huang, Min Kang, Min Yi, Hui Yang, Sibei Wu, Rensheng Wang

**Affiliations:** ^1^ Department of Oncology, Fourth Affiliated Hospital of Guangxi Medical University, Liuzhou, Gaungxi 545000, China; ^2^ Department of Radiation Oncology, First Affiliated Hospital of Guangxi Medical University, Nanning, Guangxi 530021, China; ^3^ Department of Pathology, Fourth Affiliated Hospital of Guangxi Medical University, Liuzhou, Gaungxi 545000, China

**Keywords:** Ki67, ERCC1, prognosis, nasopharyngeal carcinoma

## Abstract

This study aimed to assess the predictive value of combined Ki67 and ERCC1 in distant metastasis-free nasopharyngeal carcinoma. 334 such cases were retrospectively assessed. Immunohistochemistry was used to evaluate Ki67 and ERCC1 protein levels in tumor tissues. Associations of Ki67 and ERCC1 amounts with clinical characteristics and survival were analyzed. Medium follow-up was 48.7 months; overall survival (OS), disease-free survival (DFS), distant metastasis-free survival (DMFS), and local recurrence-free survival (LRFS) were 91.3%, 76.0%, 82.0%, and 91.9%, respectively. High Ki67 expression was found in 35.6% patients, and positively correlated with clinical- and N- staging (*P =* 0.005, *P* < 0.001); 4-year OS, DFS, and DMFS were significantly lower in the high Ki67 group than patients with low-medium expression (*P =* 0.001, *P =* 0.012, *P =* 0.007). High ERCC1 expression was found in 35.3% of patients, and positively correlated with clinical- and T- staging. Compared with low ERCC1 expression cases, 4-year OS, DFS, DMFS, and LRFS were decreased significantly in those with high levels. High Ki67 and ERCC1 levels were related to adverse prognoses of OS (HR=4.977, 95% CI 2.31–12.292, P<0.001), DFS (HR = 4.178, 95% CI 2.421–7.212, *P* < 0.001), DMFS (HR = 3.722, 95% CI 2.028–7.015, *P* < 0.001), and LRFS (HR = 3.689, 95% CI 1.423–9.566, *P =* 0.007). Compared with the low-medium Ki67 and low ERCC1 groups, no significant difference in survival prognosis was obtained in the low-medium Ki67 and high ERCC1 groups, and patients with high Ki67 and low ERCC1 levels. Combined Ki67 and ERCC1 can better predict nasopharyngeal carcinoma prognosis than individual parameters.

## INTRODUCTION

Nasopharyngeal carcinoma (NPC) is primarily found in the nasopharynx. As one of the world regions with highest incidence rates for this disease, south China has a standardized incidence rate of 20–30/100 000 [1, 2]. Non-keratinizing carcinoma (WHO 2005 classification), especially the undifferentiated type of Non-keratinizing carcinoma [3], is the major pathological type in regions with high incidence, and has better sensitivity to chemoradiotherapy. Oncogenes and tumor suppressor genes are involved in tumorigenesis and development, while DNA repair genes are involved in drug resistance [4–6]. Expression level of these protein should be considered to predict treatment responses and prognoses in NPC patients besides pathological type, clinical staging, and chemoradiotherapy. Therefore, understanding the underlying intrinsic factors would help predict the prognosis and select optimal treatments.

The Ki67 protein is closely related to mitosis and reflects the proliferation activity of malignant tumors [7], while indicating the development and prognosis of various malignant tumors such as breast, lung, urinary tract, and cervical cancers [8–11]. As an independent prognostic indicator, it is conventionally applied in the molecular classification and treatment strategies of breast cancer [12]. Regarding head and neck cancer, high expression of Ki67 affects the treatment response of oral squamous cell carcinoma [13] with high risk of recurrence of early tongue cancer [14]. However, the significance of Ki67 expression in NPC is unclear, and conclusions from few small sample studies are different [15, 16].

ERCC1 is involved in the DNA nucleotide excision repair process; it identifies and resects the damaged DNA strand. High ERCCl expression is associated with resistance to platinum-based chemotherapy [17–19]; patients with low ERCC1 expression are more likely to obtain survival benefit [18, 19]. ERCC1 expression predicts efficacy in patients with non-small cell lung cancer or head and neck squamous cell carcinoma, who have received platinum-based concurrent chemoradiotherapy; meanwhile, patients with low ERCC1 expression often show better treatment response and survival [20, 21]. Meta-analysis has shown that ERCC1 expression is associated with the efficacy of platinum-based chemoradiotherapy for head and neck squamous cell carcinoma as well as prognosis of the Asian population; meanwhile, Asians with low ERCC1 expression show better OS and PFS [22–24]. Moreover, ERCC1 level shows a correlation with objective response rate (ORR) and OS in NPC [22]. However, NPC data are from a few small sample studies, and the association of ERCC1 expression with treatment response and prognosis in NPC needs to be confirmed by additional large sample studies.

In addition, whether combined Ki67 and ERCC1 can better assess prognosis and provide clinical guidance remains unclear. This study aimed to retrospectively assess the prognostic value of combined Ki67 and ERCC1 levels and survival in the major pathologic type (non-keratinizing carcinoma) in a high-incidence area of NPC.

## RESULTS

### Cases and follow-up status

A total of 334 cases with complete data were assessed. Medium age was 48 years (17–72 years); males accounted for 73.1%, and females for 26.9%. The differentiated and undifferentiated types of Non-keratinizing carcinoma accounted for 5.1% and 94.9%, respectively. I-II stage disease accounted for 12.6%, while III-IVb was found in 87.4% of cases. Radiotherapy was performed by IMRT. A total of 3.6%, 13.5%, 39.5%, and 43.4% of patients received radiotherapy alone, platinum-based concurrent radiochemotherapy, platinum-based induction chemotherapy and concurrent radiochemotherapy, and platinum-based concurrent radiochemotherapy and adjuvant combination chemotherapy, respectively (Table 1).

**Table 1:** Patient characteristics

Characteristics	No. of patients (range)	%
Total number of patients	334	100
Age (Years)	48 (17–72)	
Gender		
Males	244	73.1
Females	90	26.9
pathological subtype of Non-keratinizing carcinoma (2005 WHO)*		
Differentiated type	17	5.1
Undifferentiated type	317	94.9
Clinical stage (AJCC 7th, 2010)#		
I	3	0.9
II	39	11.7
III	160	47.9
IV a/b	132	39.5
Primary tumor (T) stage		
T1	27	8.1
T2	55	16.5
T3	141	42.2
T4	111	33.2
Regional lymph nodes (N) stage		
N0	20	6.0
N1	116	34.7
N2	161	48.2
N3	37	11.1
Treatment received by patients		
Radiotherapy alone	12	3.6
Concurrent chemoradiation	45	13.5
Induction chemotherapy + Concurrent chemoradiation	132	39.5
Concurrent chemoradiation + Adjuvant chemotherapy	145	43.4

*According to the 2005 WHO histological classification and pathologic stage [3]

^#^According to the 2010 International Union against Cancer/American Joint Committee on Cancer (UICC/ AJCC) staging system [25] for NPC.

Medium follow-up was 48.7 months (5–72 months) and ended in December 2016. The 1-, 2-, 3-, and 4-year OS rates were 99.4%, 96.7%, 92.8%, and 91.3%, respectively; DFS rates were 94.3%, 82.3%, 79.3%, and 76.0%, respectively; DMFS rates were 95.5%, 87.7%, 84.4%, and 82.0%, respectively; LRFS rates were 98.5%, 94.6%, 93.1%, and 91.9%, respectively. The disease had progressed in 80 cases, including 50 cases with distant metastasis as the initial event, 17 with local recurrence (7 in primary lesion, 9 in regional lymph nodes, and 3 in both primary lesion and regional lymph nodes), 10 with distant metastasis combined with local recurrence, and 3 with a second primary tumor (1 each of tongue cancer, gastric cancer, and nasal sarcoma). There were 29 cases of death.

### Expression levels of Ki67 and ERCC1 in nasopharyngeal carcinoma

Of the 334 patients, low-medium Ki67 expression accounted for 64.4% (low and medium levels represented 27.5% and 36.9%, respectively), while high expression was found in 35.6%. Low expression of ERCC1 was found in 64.7% of patients, and high in 35.3%.

The expression of Ki67 was not significantly correlated with gender (χ^2^ = 1.096, *P* = 0.179), pathological subtype of Non-keratinizing carcinoma (χ^2^ = 0.302, *P* = 0.395), and T staging (χ^2^ = 7.496, *P* = 0.058), but significantly associated with clinical staging (χ^2^ = 12.714, *P* = 0.005) and N staging (χ^2^ = 28.004, *P* =< 0.001). ERCC1 expression was not significantly associated with gender (χ^2^ = 1.588, *P* = 0.128), pathological subtype of Non-keratinizing carcinoma (χ^2^ = 0.910, *P* = 0.475), and N staging (χ^2^ = 0.205, *P* = 0.977), but significantly associated with clinical staging (χ^2^ = 14.57, *P* = 0.002) and T staging (χ^2^ = 15.47, *P* = 0.002) (Table 2).

**Table 2:** Associations of Ki67 and ERCC1 levels in nasopharyngeal carcinoma with clinical characteristics

Characteristics	Ki67 *n =* 334		χ^2^ value	*P*	ERCC1 *n =* 334		χ^2^ value	*P*
	Low-medium expression (%)	High expression (%)			Low expression (%)	High expression (%)		
Total cases	215	119			216	118		
Gender								
Male	153 (71.2%)	91 (76.5%)	1.096	0.179	147 (70.7%)	97 (77.0%)	1.588	0.128
Female	62 (28.8%)	28 (23.5 %)			61 (29.3%)	29 (23.0%)		
Non-keratinizing carcinoma								
Differentiated type	12 (5.6%)	5 (4.2%)	0.302	0.395	10 (4.8%)	7 (5.6%)	0.910	0.475
Undifferentiated type	203 (94.4%)	114 (95.8%)			198 (95.2%)	119 (94.4%)		
Clinical staging								
Stage I	3 (1.4%)	0 (0.0%)	12.714	0.005	2 (1.0%)	1 (0.8%)	14.576	0.002
Stage II	33 (15.3%)	6 (5.0%)			35 (16.8%)	4 (3.2%)		
Stage III	105 (48.8%)	55 (46.2%)			96 (46.2%)	64 (50.8%)		
Stage IV a/b	74 (34.4%)	58 (48.7%)			75 (36.1%)	57 (45.2%)		
T staging								
T1	21 (9.8%)	6 (5.0%)	7.496	0.058	18 (8.7%)	9 (7.1%)	15.074	0.002
T2	38 (17.7%)	17 (14.3%)			46 (22.1%)	9 (7.1%)		
T3	95 (44.2%)	46 (38.7%)			85 (40.9%)	56 (44.4%)		
T4	61 (28.4%)	50 (42.0%)			59 (28.4%)	52 (41.3%)		
N staging								
N0	15 (7.0%)	5 (4.2%)	28.004	<0.001	12 (5.8%)	8 (6.3%)	0.205	0.977
N1	95 (44.2%)	21 (17.6%)			73 (35.1%)	43 (34.1%)		
N2	87 (40.5%)	74 (62.2%)			101 (48.6%)	60 (47.6%)		
N3	18 (8.4%)	19 (16.0%)			22 (10.6%)	15 (11.9%)		

### Ki67 expression and nasopharyngeal carcinoma prognosis

**Figure 1:**
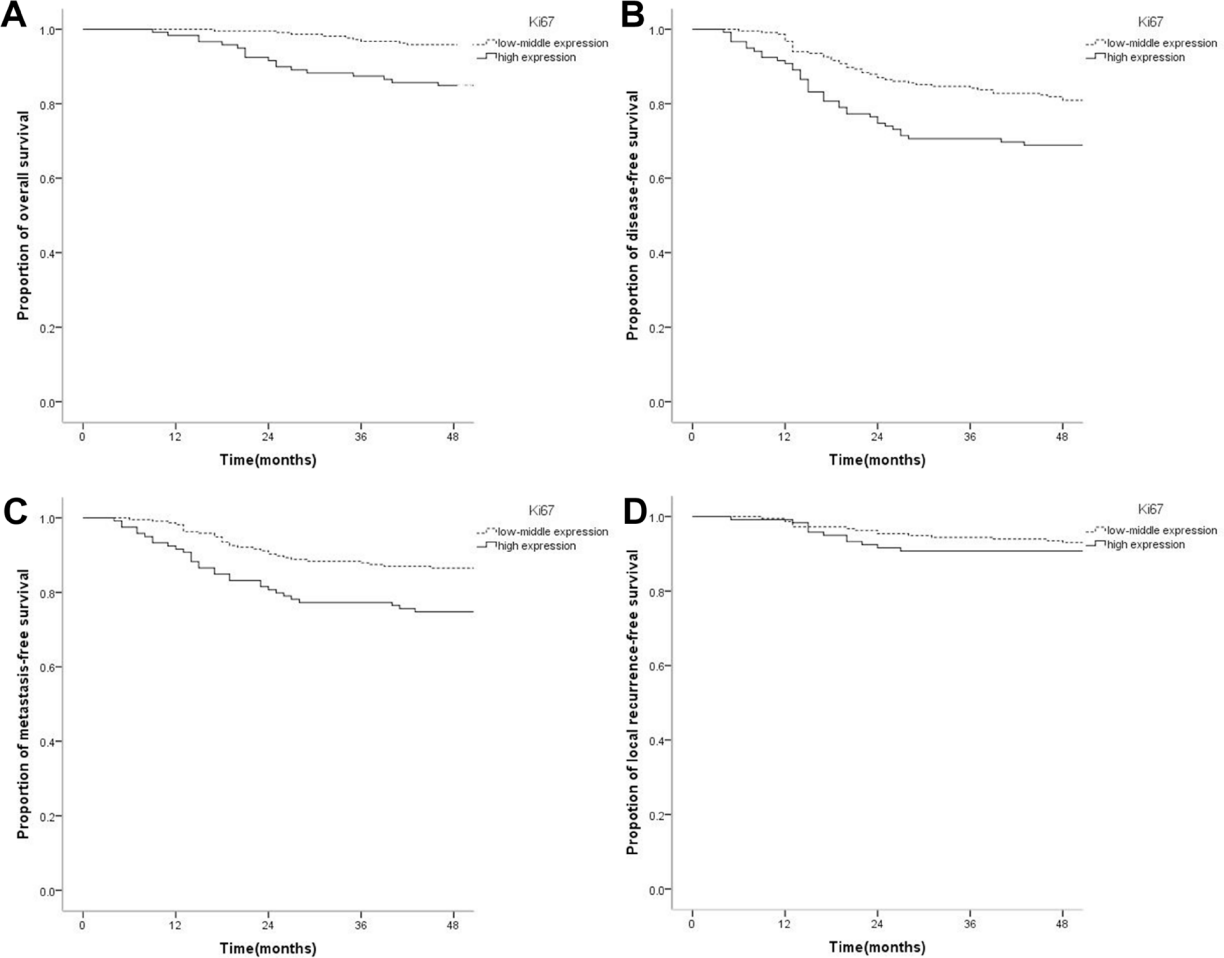
Kaplan-Meier survival curves of nasopharyngeal carcinoma patients with low-medium and high Ki67 levels (**A**) The overall survival rate of patients with high Ki67 expression was significantly lower than that of the low-medium expression group (*P* = 0.001). (**B**) The disease-free survival rate of patients with high Ki67 level was significantly lower than that of the low-medium expression group (*P* = 0.012). (**C**) The distant metastasis-free survival rate of patients with high Ki67 expression was significantly lower than that of the low-medium expression group (*P* = 0.007). (**D**) The local recurrence-free survival rate was not significantly different between the high and low Ki67 expression groups (*P* = 0.547).

OS, DFS, and DMFS in patients with high Ki67 expression were significantly lower than those with low-medium expression. 4-year OS rates in the low-medium and high expression groups were 94.9% and 84.9%, respectively (*P* = 0.001); 4-year DFS rates were 80.0% and 68.9%, respectively (*P* = 0.012); 4-year DMFS rates were 86.0% and 74.8%, respectively (*P* = 0.007). There was no statistically significant difference in LRFS between the high and low-medium Ki67expression groups: 4-year LRFS in the low-medium and high expression groups were 92.6% and 90.8%, respectively (*P* = 0.547) (Figure 1). However, OS, DFS, DMFS, and LRFS were similar in the low and medium Ki67 expression groups. 4-year OS rates in the low and medium expression groups were 95.7% and 94.3%, respectively (*P* = 0.449); 4-year DFS rates were 84% and 77.2%, respectively (*P* = 0.136); 4-year DMFS rates were 89.1% and 84.6%, respectively (*P* = 0.266); LRFS rates were 93.5% and 92.7%, respectively (*P* = 0.515). High Ki67 expression was correlated with adverse prognosis of OS (HR = 3.155, 95% CI 1.489–6.683, *P* = 0.003), DFS (HR = 1.734, 95% CI 1.119–2.695, *P* = 0.014), and DMFS (HR = 1.974, 95% CI 1.190–3.274, *P* = 0.008) in NPC, but did not significantly increase LRFS risk (HR = 1.266, 95%CI 0.587–2.728, *P* = 0.547). It means that high Ki67 expression could predict adverse prognosis of OS, DFS and DMFS.

### ERCC1 expression and nasopharyngeal carcinoma prognosis

**Figure 2:**
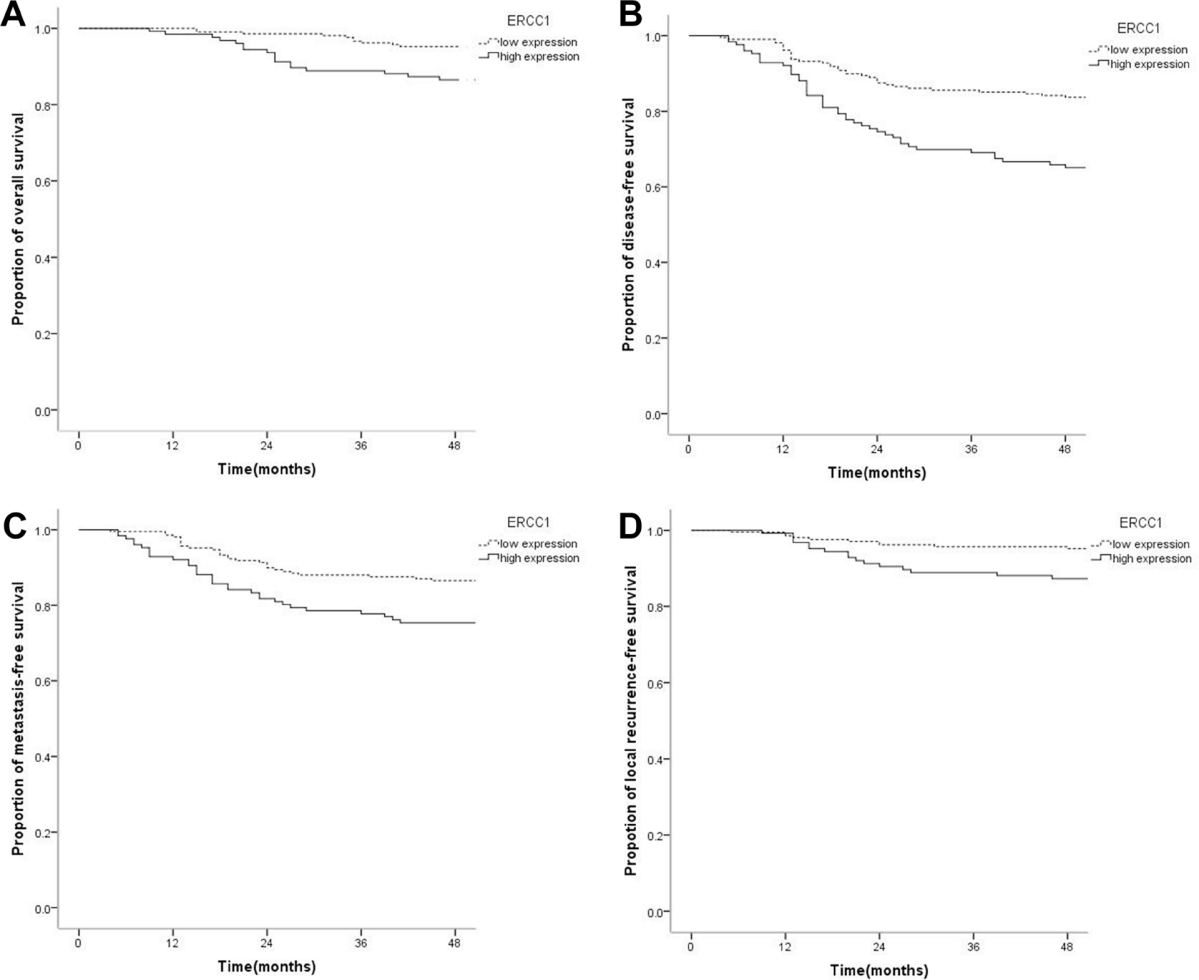
Kaplan-Meier survival curves of nasopharyngeal carcinoma patients with low ERCC1 level and those with high expression (**A**) The overall survival rate of patients with high expression of ERCC1 was significantly lower than that of the low expression group (*P* = 0.013). (**B**) The disease-free survival rate of patients with high ERCC1 expression was significantly lower than that of the low expression group (*P* < 0.001). (**C**) The distant metastasis-free survival rate of patients with high ERCC1 amounts was significantly lower than that of the low expression group (*P* = 0.005). (**D**) The local recurrence-free survival rate of patients with high ERCC1 expression was significantly lower than that of the low expression group (*P* = 0.005).

Compared with the low expression group, OS, DFS, MDFS, and LRFS rates in the high ERCC1 expression group were decreased significantly. Specifically, 4-year OS in the low and high expression groups were 94.2% and 86.5%, respectively (*P* = 0.013); 4-year DFS rates were 83.2% and 64.3%, respectively (*P* < 0.001); 4-year DMFS rates were 86.5% and 74.6%, respectively (*P* = 0.005); 4-year LRFS rates were 95.2% and 86.5%, respectively (*P* = 0.005) (Figure 2). High ERCC1 expression was associated with adverse prognosis of, with risks of OS (HR = 2.409, 95%CI 1.147–5.058, *P* = 0.020), DFS (HR = 2.381, 95%CI 1.530–3.705, *P* < 0.001), DMFS (HR = 2.039, 95%CI 1.228–3.386, *P* = 0.006), and LRFS (HR = 2.909, 95%CI 1.332–6.354, *P* = 0.007) increased obviously.

### Combined Ki67 and ERCC1 expression and survival of nasopharyngeal carcinoma

Of the 334 cases of NPC, the proportion with concurrent high Ki67 and ERCC1 expression was 13.5% (45/334); low-medium Ki67 expression combined with high ERCC1 expression was found in 24.0% (80/334). 21.6% (72/334) of patients had high Ki67 expression combined with low ERCC1 expression, while 41.0% (137/334) showed low-medium Ki67 and low ERCC1 amounts.

**Figure 3:**
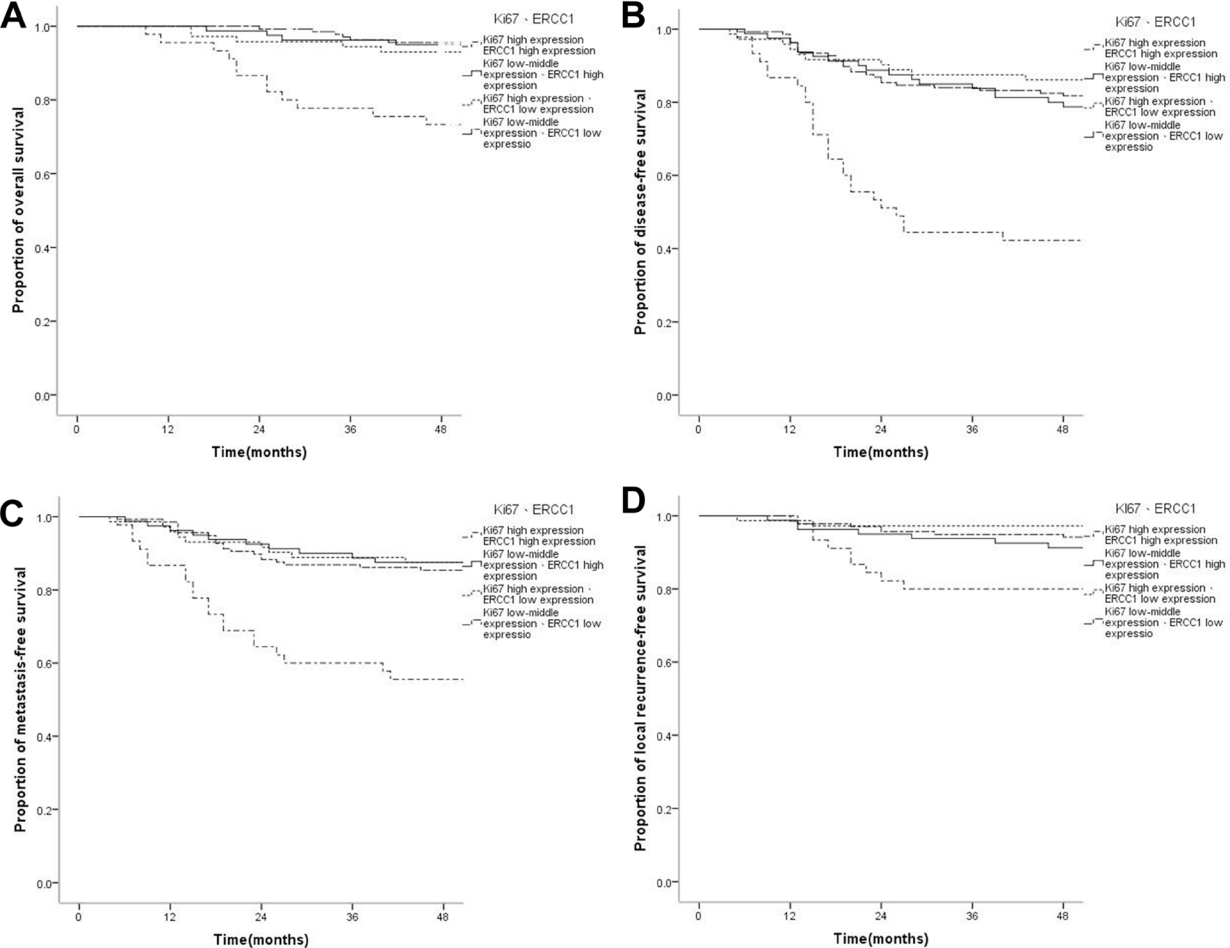
Kaplan-Meier survival curves of patients with nasopharyngeal carcinoma displaying different combinations of Ki67 and ERCC1 expressions (**A**) The overall survival rate of patients with concurrent high ERCC1 and Ki67 levels was significantly lower than those of the low-medium Ki67/high ERCC1, high Ki67/low ERCC1, and low-medium Ki67/low ERCC1 groups (*P* < ss0.001); there were no statistically significant differences in overall survival rate among the low-medium Ki67/high ERCC1, high Ki67/low ERCC1, and low-medium Ki67/low ERCC1 groups (*P* = 0.878).(**B**) The disease-free survival rate of patients with concurrent high ERCC1 and Ki67 levels was significantly lower than those of the low-medium Ki67/high ERCC1, high Ki67/low ERCC1, and low-medium Ki67/low ERCC1 groups (*P* < 0.001); however, there were no statistically significant differences in disease-free survival among the low-medium Ki67/high ERCC1, high Ki67/low ERCC1, and low-medium Ki67/low ERCC1 groups (*P* = 0.581). (**C**) The distant metastasis-free survival rate of patients with concurrent high ERCC1 and Ki67 levels was significantly lower than those of the low-medium Ki67/high ERCC1, high Ki67/low ERCC1, and low-medium Ki67/low ERCC1 groups (*P* < 0.001); however, there were no statistically significant differences in the distant metastasis-free survival rate among the low-medium Ki67/high ERCC1, high Ki67/low ERCC1, and low-medium Ki67/low ERCC1 groups (*P* = 0.416). **(D**) The local recurrence-free survival rate of patients with concurrent high ERCC1 and Ki67 levels was significantly lower than those of the low-medium Ki67/high ERCC1, high Ki67/low ERCC1, and low-medium Ki67/low ERCC1 groups (*P* = 0.005); however, there were no statistically significant differences in local recurrence-free survival among the low-medium Ki67/high ERCC1, high Ki67/low ERCC1, and low-medium Ki67/low ERCC1 groups (*P* = 0.266).

OS, DFS, DMFS, and LRFS rates in cases with concurrent high Ki67 and ERCC1 expression levels were significantly lower than those of other groups. Specifically, 4-year OS rates were 73.3%, 95.0%, 93.1% and 94.2%, respectively, in patients with concurrent high ERCC1 and Ki67 levels, low-medium Ki67 and high ERCC1 levels, high Ki67 and low ERCC1 amounts, and low-medium Ki67 and low ERCC1 amounts (*P* < 0.001); 4-year DFS rates were 42.2%, 77.5%, 86.1% and 81.0%, respectively (*P* < 0.001); 4-year DMFS rates were 55.6%, 86.3%, 87.5%, 85.4%, respectively (*P* < 0.001); 4-year LRFS rates were 80.0%, 90.0%, 97.2%, 94.2%, respectively (*P* = 0.005). There were no statistically significant differences in OS, DFS, DMFS, and LRFS rates among patients with low-medium Ki67 and high ERCC1 levels, high Ki67 and low ERCC1 amounts, and low-medium Ki67 and low ERCC1 levels (*P* = 0.878, *P* = 0.581, *P* = 0.416, *P* = 0.266) (Figure 3). Compared with the low-medium Ki67 and low ERCC1 group, patients with concurrent high Ki67 and ERCC1 levels showed significantly increased risks of OS (HR = 4.977, 95% CI 2.31–12.292, *P* < 0.001), DFS (HR = 4.178, 95% CI 2.421–7.212, *P* < 0.001), DMFS (HR = 3.722, 95% CI 2.028–7.015, *P* < 0.001), and LRFS (HR = 3.689, 95% CI 1.423–9.566, *P* = 0.007) in NPC, which were closely correlated with adverse prognosis; however, no statistically significant difference in the risk of survival prognosis was obtained in the low-medium Ki67 and high ERCC1 group, and patients with high Ki67 and low ERCC1 levels (*P* > 0.05) (Table 3). Multivariate analysis was performed by Cox risk proportion model, only concurrent high expression of Ki67 and ERCC1 increased the risk of every survival endpoint (*P* < 0.048) (Table 4). These results indicated that only concurrent high expression of Ki67 and ERCC1 was closely correlated with unfavorable prognosis.

**Table 3:** Survival risks compared with the low-medium Ki67 and low ERCC1 group

Variable	HR	95%CI		*P*
		Lower	Upper	
OS				
Ki67 high, ERCC1 high	4.997	2.031	12.292	< 0.001
Ki67low-medium, ERCC1 high	0.870	0.262	2.890	0.820
Ki67 high, ERCC1low-medium	1.259	0.412	3.851	0.686
DFS				
Ki67 high, ERCC1 high	4.178	2.421	7.212	< 0.001
Ki67low-medium, ERCC1 high	1.183	0.649	2.158	0.583
Ki67 high, ERCC1low-medium	0.723	0.349	1.500	0.384
MDFS				
Ki67 high, ERCC1 high	3.722	2.028	7.015	< 0.001
Ki67low-medium, ERCC1 high	0.933	0.447	1.946	0.853
Ki67 high, ERCC1low-medium	0.853	0.388	1.874	0.692
LRFS				
Ki67 high, ERCC1 high	3.689	1.423	9.566	0.007
Ki67low-medium, ERCC1 high	1.736	0.652	4.626	0.270
Ki67 high, ERCC1 low-medium	0.473	0.101	2.229	0.344

**Table 4:** Multivariate analysis of the Cox risk ratio model for survival endpoints of combined Ki67 and ERCC1 expression in non-keratinizing nasopharyngeal carcinoma

	OS			DFS			DMFS			LRFS		
	HR	95%CI	*P*	HR	95%CI	*P*	HR	95%CI	*P*	HR	95%CI	*P*
Gender	0.290	0.087–0.965	0.044	0.858	0.510–1.444	0.565	0.872	0.483–1.573	0.649	0.666	0.250–1.722	0.416
Pathology subtype	0.833	0.111–6.273	0.859	1.028	0.372–2.840	0.584	1.020	0.315–3.299	0.974	0.742	0.099–5.552	0.772
Clinical stage	0.615	0.206–1.840	0.385	1.141	0.632–20.59	0.662	0.801	0.379–1.693	0.561	1.650	0.599–4.545	0.332
T stage	1.751	0.787–3.898	0.170	1.238	0.847–1.810	0.448	1.664	0.985–2.813	0.057	0.983	0.538–1.798	0.956
*N* stage	1.764	0.944–3.131	0.053	1.612	1.137–2.285	0.014	1.915	1.268–2.891	0.002	1.298	0.727–2.318	0.378
Combined Ki67 and ERCC1 expression												
Ki67 high, ERCC1 high	3.532	1.395–9.178	0.010	2.961	1.675–5.234	<0.001	2.563	1.324–4.858	0.005	2.696	1.008–7.215	0.048
Ki67low-medium, ERCC1 high	0.755	0.225–2.530	0.648	1.110	0.606–2.035	0.735	0.874	0.415–1.837	0.722	1.612	0.600–4.328	0.343
Ki67 high, ERCC1low-medium	0.964	0.312–2.976	0.949	0.562	0.269–1.172	0.124	0.636	0.287–1.407	0.264	0.392	0.083–1.857	0.238
Low-medium Ki67 and low ERCC1	Reference group											

## DISCUSSION

The combination therapy with IMRT as the major therapy has dramatically improved the prognosis of NPC patients; metastasis is the main manifestation of failed treatment [26, 27]. In this study, 87.4% of patients had local advanced disease, and 4-year OS and LRFS rates were 91.3% and 91.9%, respectively, for a DMFS rate of 82.0%, achieving better overall survival and local control. Distant metastasis was the main cause of disease progression, consistent with previous reports [26, 27]. With inherent tumor parameters, some patients with the same clinical characteristics and treatment show different treatment responses and prognoses [26]. Therefore, understanding potential biological indicators and a more accurate prediction of NPC prognosis might contribute to individual treatment options.

It is convenient to detect Ki67 and ERCC1 expression levels in primary tumor tissues as biological prognostic indicators. In this study, patients with NPC expressed Ki67 and ERCC1 at various degrees. Ki67 expression can highly reflect the proliferative activity of malignant tumors [7]; however, its threshold for clinical significance remains unclear. The 14% threshold recommended by common sense remains controversial even in breast cancer [12]. The limited available small sample studies of NPC involving Ki67 expression have yielded discrepant conclusions due to different thresholds used. Gene et al. [15] evaluated 35 NPC cases, and found that Ki67 < 10% indicates longer survival (*P* = 0.03), but Ki67 expression was not significantly associated with T/N and clinical staging. Ma et al. [16] used 60% as a threshold for high Ki67 expression, and found that Ki67 expression is not significantly associated with clinical staging and survival prognosis. In this study, OS, DFS, DMFS, and LRFS rates between patients with Ki67 ≤ 25% (low expression) and those with Ki67 > 25%∼50% (median expression) were not significantly different (*P* = 0.449, *P* = 0.136, *P* = 0.266, *P* = 0.515). For the high expression group (Ki67 > 50%), OS, DFS, and DMFS rates decreased significantly compared with the low-medium expression group (*P* = 0.001, *P* = 0.012, *P* = 0.007), with the corresponding risks of adverse prognosis increasing significantly (*P* = 0.003, *P* = 0.014, *P* = 0.008), corroborating the findings by Shi et al. [28], who used 50% as observation threshold. Due to sample size difference, the high expression rate of Ki67 in this study was 35.6%, which was lower than that reported by Shi et al. (54.5%, 30/55) [28]; meanwhile, Ki67 expression was significantly correlated with clinical staging (*P* = 0.005) and N staging (*P* < 0.001). Thus, selection of an appropriate threshold based on sample size is key to effectively investigate the predictive role of Ki67 in NPC; Ki67 > 50% used as a better observation threshold could reveal adverse prognosis in NPC. Observations with larger samples could help clarify the uncertainty of the association of Ki67 expression with T staging observed in this study (*P* = 0.058).

We also assessed ERCC1, whose high expression in locally advanced NPC was demonstrated in 14.5%-74% patients [22, 29–31]. As shown above, high expression of ERCC1 was found in 35.6% of all patients (including 12.8% of early cases). A meta-analysis [22–24] showed that high ERCC1 expression is related to adverse prognosis of OS in NPC (HR = 2.72, 95% CI 1.79–4.13, *P* < 0.001), but not significantly related to the clinical characteristics DFS, DMFS, and LRFS; however, sample sizes of individual studies were small, and conclusions were not consistent. Huang et al. [30] reported that 5-year DMFS of patients with high ERCC1 expression is significantly lower than that of the low expression group (51.3% and 73.5%, respectively, *P* = 0.037). Meanwhile, Zhang Z et al. [32] revealed that high ERCC1 expression is associated with higher local recurrence rate (29.4% vs. 12.5%, *P* = 0.041). However, Liang et al. [31] assessed 76 cases of NPC, and demonstrated that although ERCC1 expression is not significantly correlated with gender and N staging, it is significantly associated with T staging, clinical staging, and OS (*P* < 0.05), corroborating our findings. However, in this larger sample studies, OS, DFS, DMFS, and LRFS rates in patients with high ERCC1 expression were shown to decrease significantly, while the risk of adverse prognosis increases markedly, compared with those with low expression.

In this study, high expression of Ki67 or ERCC1 alone indicated adverse prognosis of NPC. However, combined analysis found no statistically significant differences in OS, DFS, DMFS, and LRFS rates as well as corresponding prognostic risks between the patients with Ki67 and ERCC1 cross-expression, and those with low-medium Ki67 expression combined with low ERCC1 levels (*P* > 0.05). On the one hand, tumor cells are in the proliferation period when Ki67 is highly expressed. Highly proliferative cells often are more sensitive to drugs and radiation, which results in better therapeutic response [32]. Koelbl et al. [33] reported a complete remission (CR) rate of preoperative chemoradiotherapy in OSCC patients with high Ki67 expression of 43.5%. This was significantly higher than values obtained in the low (26.1%) and medium (30.9%) expression groups. In this study, the high and low-medium Ki67 expression groups showed no statistically significant difference in LRFS of NPC (*P* = 0.547), and local recurrence risk did not increase significantly (HR = 1.266, 95% CI 0.587–2.728, *P* = 0.547). On the other hand, tumors with high ERCC1 expression have better ability of damage repair, which is associated with resistance to platinum radiotherapy. The therapeutic response of NPC is therefore worse [31, 34]. Sun et al. [34] assessed 118 cases of NPC who received radiation therapy alone, and found that the LRFS rate in patients with high ERCC1 expression is significantly lower than that of the low expression group (70% vs 86%, *P* = 0.046). Liang et al. [31] evaluated 76 NPC cases with concurrent chemoradiotherapy, and demonstrated that the response rate of patients with positive ERCC1 expression is significantly lower than that of the negative expression group (75% vs 97.7%, *P* = 0.008). Therefore, although NPC with high Ki67 and low ERCC1 levels has higher risk of cell proliferation, it also presents increased sensitivity to chemoradiotherapy, which can improve prognosis. However, in patients with low-medium Ki67 and high ERCC1 levels, although there is a certain degree of resistance to chemoradiotherapy, the risk of proliferation for the tumor itself is not high, which can yield relatively better prognosis. With both Ki67 and ERCC1 highly expressed, and the patients having both high risk of tumor proliferation and resistance to treatment, the risk of adverse prognosis may increase significantly.

In this study, 82.9% of patients received concurrent chemoradiotherapy and chemotherapy. Under sufficient treatment, compared with patients with low-medium Ki67 and low ERCC1 levels, OS, DFS, DMFS, and LRFS rates in patients with high Ki67 and low ERCC1 amounts did not decrease significantly, with the corresponding prognostic risk not statistically significant (*P* > 0.05). There were no statistically significant differences in survival indicators for patients with low-medium Ki67 and high ERCC1 levels after treatment (*P* > 0.05); indeed, concurrent high expression of Ki67 and ERCC1 was closely correlated with adverse prognosis (*P* < 0.007), and OS, DFS, DMFS, and LRFS rates decreased significantly (*P* < 0.005). Thus, combined Ki67 and ERCC1 can predict survival prognosis more effectively and guide treatment options. Systematic treatment of sufficient intensity can improve the prognosis of patients with high Ki67 levels combined with low ERCC1 expression, yielding better survival. However, patients with low-medium Ki67 expression had limited treatment sensitivity as low risk cases; therefore, they might be exempted from high-intensity treatment.

This study had some limitations. First, due to limited data in this retrospective analysis, we did not assess the efficacy of Ki67 and ERCC1 as well as chemoradiotherapy, or verify the impacts of Ki67 can ERCC1 on radiation and drug sensitivity. Insufficient follow-up time and small sample size were additional shortcomings. Longer follow-up and/or prospective studies with larger sample size would be helpful to further confirm the actual significance of Ki67 and ERCC1 expression in NPC. Moreover, combination analysis of Ki67 and ERCC1 could guide prognosis prediction and treatment options for NPC.

High expression levels of Ki67 and ERCC1 are associated with adverse prognosis of NPC. Combined analysis can be more effective in predicting survival prognosis. Ki67 and ERCC1 highly and concurrently expressed result in significantly decreased OS, DFS, DMFS, and LRFS rates, with increased risk of adverse prognosis, suggesting this combination can indicate adverse prognosis more sensitively; reasonable treatment may improve the survival of patients with cross-expression of Ki67 and ERCC1.

## MATERIALS AND METHODS

### Clinical data

362 NPC patients were selected from the Fourth Affiliated Hospital of Guangxi Medical University from January 2011 to December 2014. Inclusion criteria were: 1) no distant metastasis or previous treatment; 2) pathology of non-keratinizing carcinoma according to the 2005 WHO pathological classification [3]; 3) defined stage according to seventh edition of UICC/AJCC clinical staging standard [25]; 4) IMRT-based radiotherapy or Platinum-based chemoradiotherapy. The study was approved by the Ethics Committee of our Hospital, and informed consent was waived.

### Immunohistochemistry

All tissue samples are gained from the nasopharyngeal biopsy by the nasopharyngeal lens in the diagnose and immunohistochemistry should be finished before the therapy.

Tissue sections were dewaxed and hydrated, and placed in 0.01 mol/L citrate buffer at pH 6.0 for high temperature and high pressure antigen retrieval. The sections were washed with PBS for 5 min, and 3% H_2_O_2_ was added for 10 min at room temperature. Next, the sections were washed with PBS for 5 min (three times) before addition of primary antibodies raised against Ki67 and ERCC1, respectively, for overnight incubation at 4°C. Subsequently, the sections were incubated with secondary antibodies for 15 min at room temperature. After washing with PBS as above, the DAB reagent was added for color development, and counterstaining was carried out by hematoxylin staining. All antibodies and kits were purchased from Beijing Zhongshan Biotechnology Co., Ltd (China).

### Immunohistochemistry data interpretation

Independent assessments of the results for immunohistochemical staining of ERCC1 and Ki67 were performed by two blinded pathologists.

Ki67 positive cells were graded as: < 10%, grade 0; 10%∼25%, grade 1 (+); > 25%∼50%, grade 2 (++); > 50%, grade 3 (+++). Furthermore, ≤ 25% was considered low expression, >25%∼50% medium level, and > 50% high expression.

ERCC1 positive cells were graded as: < 5%, grade 0; 5%∼25%, grade 1; 26%∼50%, grade 2; 5l%∼75%, grade 3; > 75%, grade 4. Staining grades included: 0, no staining; 1, weakly positive staining; 2, medium staining; 3, strong staining.

Based on the sum of the two results: a total score of 0∼1 represented no staining (-), 2∼3 weakly positive (+), 4∼5 medium positive (++), and 6∼7 strongly positive (+++); meanwhile, (-∼+) was considered low expression, and (++∼+++) indicated high expression.

### Statistical analysis

Statistical analyses were performed with SPSS19.0 (SPSS, USA). Categorical data were descriptively analyzed; the associations of Ki67 and ERCC1 expression levels with clinical characteristics were assessed by χ^2^ test. Overall survival (OS), disease-free survival (DFS), distant metastasis-free survival (DMFS) and local recurrence-free survival (LRFS) were assessed. The Kaplan-Meier method and log-rank test, as well as the cox risk ratio model were used to perform correlation analyses of survival and prognosis. Two sided *P* < 0.05 was considered statistically significant.

## Abbreviations

OS: overall survival
DFS: disease-free survival
DMFS: distant metastasis-free survival
LRFS: local recurrence-free survival
NPC: Nasopharyngeal carcinoma
ORR: objective response rate
